# Notes to a young epidemiologist

**DOI:** 10.1017/ash.2022.352

**Published:** 2022-12-15

**Authors:** Richard P. Wenzel

**Affiliations:** Virginia Commonwealth University Health Systems, Richmond, Virginia

Reflecting on my career and the experiences that most lift my spirits, I conclude it was the time I spent with pre and postdoctoral students. At the bedside, in the classroom, or one-on- one with a learner, I loved the exchanges with learners. This preference is surely a window into my perspectives. Learners often became special friends and professional relatives. I followed their career trajectories with excitement, considered ways in which many succeeded, and summarized key themes, a few of which are contained herein.

## A Mentor

Early in Homer’s epic poem, *The Odyssey,* the hero prepares to depart for the Trojan War, imploring his elderly friend, Mentor, to oversee the care of his infant son, Telemachus. It was an awesome responsibility—the war lasted 10 years, and it took an additional 10 years for Odysseus to return to Ithaca.

Today, the term mentor indicates a generous senior adviser guiding a less experienced individual, imparting wisdom and support. As you begin your career in epidemiology, I suggest searching carefully for a true mentor, someone committed to your success and advancement in the field. A valued mentor volunteers for the task, expects no advantages for herself, and remains focused on the career of the younger colleague. She celebrates the successes of the learner and strongly encourages autonomy and creativity of the mentee, continually reinforcing the activities that promote advancement in our discipline.

Additional duties of an effective mentor include shielding the mentee from accepting membership on too many committees or requests to perform too numerous manuscript reviews or being distracted by faculty invitations to join additional projects. In short, mentors protect the time of the younger person and free the younger person to thrive.

In his February 5, 1676, letter to British scientist and rival, Robert Hooke, Isaac Newton said, “If I have seen further, it is by standing on the shoulders of giants.” Mentors need not be giants, but they must offer their shoulders freely to young learners scanning the horizon.

## The Questions

Most professionals seek to master their skills in a field. In epidemiology, measures of success include performance and publication of important studies. In the academic world, this is essential. Of course, in that quest, newcomers should pursue an area that intrigues them. But my suggestion is this: At the beginning, frame your interest in the form of a question. What do you want to know? Is the question an important one? Will it be unique? Will the insights from the study advance our understanding of health and disease in a society? Or will it merely confirm earlier reports? Will your protocol properly address the question, and are the metrics appropriate?

Do not seek a safe pathway, one with few challenges. Instead take on a difficult quest, one that carries risk, but one that might make a difference in current thinking about the field. You should be quick to ask how to determine whether the question is important, unique, but still amenable to study? These questions should be addressed in critical discussions with an experienced mentor. Refinements to the question may well emerge and lead invariably to a more difficult phase of planning. What are the current assumptions behind the question and the approach to know the answer? Can these be challenged? As you identify the assumptions, write them down, and imagine exploring each repeatedly during the project, again, with the help of respected, open-minded colleagues and your mentor. In your career you will be judged not by your answers, but by your questions. Were they probing, did any challenge the status quo? Did they make a difference?

## Statistics

Success in epidemiology requires continued learning, including increased skills in assessing the scientific literature. A key skill will be your fluency in understanding statistics, their proper use, and knowledge of the underlying assumptions of statistical formulas and models. As mathematical models and artificial intelligence grow in importance, more statistical skills will be required. Do not get left behind.

I am not suggesting that you need a formal degree in statistics or have to memorize statistical formulas, write statistical programs, or examine large data sets. But you do have to know which tests are optimal to employ and the strengths and shortcomings of options.

Learning this could be a formal process in class work. However, I emphasize the value of ongoing relationships with statisticians over the periodic request for statistical help with end-of-study data. This entails day-to-day collegiality, preferably with complementary faculty assignments in both academic departments. Ideally, there would be shared space with your statistical colleagues to promote discussions.

The research protocol—the question—should be discussed with the statistician before the work is begun. In terms of manuscripts, view the statistician as a coauthor, not someone acknowledged for occasional data-crunching assistance. Your continuing education program begins with a meaningful partnership and commitment to the mutual career growth of a statistician.

## Broad networking

Mastering the field requires you to remain curious, to continually look at the challenges in epidemiology, to discuss and debate the approaches to solutions. Successful people accept debate comfortably, welcome differences of opinion, and remain open to challenge. The best approach is to surround yourself with colleagues and friends who remain animated about the field, who love ideas, who can challenge freely their own assumptions and current dogma, then ask why they should accept what is considered common sense. I have suggested that a critical first partner in the network is the statistician.

One should view the network, however, as a potentially larger team of interested colleagues—at your own institution or outside—who have varying perspectives that may broaden the impact of your study questions. Those on the front lines of medical care, medical historians, anthropologists, or economists might weigh in with some interesting implications of the study question you may not have considered. An overarching principle is that you contribute to the members in the network. How can your partnership enrich their careers? Recognize that their insights may be the spark enlivening a good study with a narrow focus to one with sweeping perspectives and novel implications for the interface of health and society.

## View the trajectory

Periodically, it is useful to review what you have accomplished in the past 3 months. Is the project moving ahead as initially thought? Has a manuscript been submitted as planned initially? What barriers are there to time management? Is there anything you are doing yourself that unwittingly creates a barrier? Are you still excited about the work you are doing? Meet with your mentor to discuss the career trajectory. A quarterly review is a worthwhile exercise. Take notes. And like the Roman god of transitions, Janus, look back, but also look forward.

Medicine and public health are undergoing major disruptions, and at times it may seem chaotic. The recent coronavirus disease 2019 (COVID-19) pandemic, the exponential growth in technology, and the financial pressures on academic centers have been disruptive. Importantly, however, opportunities emerge out of chaos. Look around. Although many choose to hunker down, keep your eyes open for the novel pathways that have opened. The field is not static but dynamic. Have a prepared mind, the iconic microbiologist Pasteur said. Learn to be comfortable with uncertainty and change. Plan for it. Hope for it. Embrace it. The future is exciting.

